# Re: Molecular testing to deliver personalised chemotherapy recommendations

**DOI:** 10.1186/s12916-023-03036-w

**Published:** 2023-09-07

**Authors:** Peter Gibbs, Wei Hong, Jeanne Tie

**Affiliations:** Division of Personalised Oncology, Walter and Eliza Hall Institute, Parkville, Melbourne, Australia

**Keywords:** Biomarker, ctDNA, Minimal residual disease

## Abstract

**Background:**

There is an increasing focus over time on the discovery and validation of biomarkers in cancer medicine, which can inform the identification of patients that are most likely to benefit from treatment, which therapy is most likely to be effective, and treatments that may not be safe.

**Body:**

Creating the necessary evidence base for biomarker-informed management is a different challenge to developing a new therapy, and many biomarkers have been adopted into routine clinical practice without phase III randomised studies where the primary endpoint was to evaluate the direct impact of a biomarker-informed approach. This has generated a robust discussion in the research and clinical community regarding the most appropriate trial methodologies for biomarker validation, and the level of evidence required to support the incorporation of individual biomarker-driven approaches as a standard of care. This ongoing debate is key to optimising clinical trial design and ultimately delivering the best possible care to patients in an environment increasingly focused on personalised and patient-focused management.

**Conclusion:**

Ongoing deliberation as to the optimal design of biomarker-driven clinical trials is critical to informing future clinical trial design and will ultimately greatly benefit patients and the clinicians that care for them.

We read with interest the interesting paper by Olivier and Prasad [1]. Their deliberations on some of the limitations of current trials of biomarker-informed patient management are particularly timely, given the rapidly emerging field of prognostic marker-guided decision-making in the adjuvant setting. They outline some concerns with the current directions regarding the real-world integration of biomarker-informed precision medicine and provide some suggested ways forward. As a robust discussion of these issues can catalyse progress in trial design and interpretation, here, we take the opportunity to respond to Olivier and Prasad.

We do concur with the authors that overtreatment in early-stage cancer currently presents a major challenge, given a prior focus on trialling new agents in broad patient populations, with limited progress in understanding which subset(s) of patients have improved outcomes. Stage II colon cancer adjuvant chemotherapy is an excellent example, where the most optimistic interpretation of trial data suggests only 1 in 20 derive a survival benefit [2]. At the same time, there is arguably under treatment, as recurrences do occur in stage II colon cancer that were not treated. For stage II colon cancer, and broadly across solid cancer types, reliable biomarkers of minimal residual disease are desperately needed, with circulating tumour DNA (ctDNA) considered the most promising [3].

## The DYNAMIC study in stage II colon cancer

Ultimately, the DYNAMIC trial [4] was a positive study, ctDNA-guided management being non-inferior to standard management with respect to 2-year recurrence-free survival (RFS). There was also significantly less chemotherapy use (a key secondary end point). Unlike Olivier and Prasad, we would suggest that the interpretation of the clinical relevance of this data is best left to expert consensus, rather than relying on social media commentary.

Olivier and Prasad speculate that in any real-world application of ctDNA the benefit of “sparing chemotherapy would evaporate,” as many high-risk patients may still be treated even if ctDNA negative. This statement erroneously implies that the end goal is treating less patients, rather the clear aim of personalised medicine is to ensure that more of those likely to benefit (e.g. ctDNA positive) are treated, and those with only a remote likelihood of benefit (and definite adverse event risk and substantial costs) can comfortably avoid treatment.

Particularly notable is that treatment in ctDNA-positive patients appears highly effective. The high 2-year RFS seen in treated ctDNA-positive patients is in stark contrast to the very high recurrence rate in observational series. Furthermore, direct evidence of chemotherapy activity in this population is ctDNA clearance in 34 of 39 (87%) patients [5]. Similar high clearance rates have been reported in an independent observational study [6]. Overall, this data suggests that ctDNA as well as being a powerful prognostic marker may also be a predictive marker of treatment benefit.

Even if all T4 ctDNA-negative patients continued to receive adjuvant chemotherapy, they represent a minority of currently treated patients. T3 disease, which accounted for 85% of the DYNAMIC cohort, made up 66% (27 of 41) of those treated from the combined T3 and T4 population, and it is this dominant T3 population where there is the greatest risk of over-treatment. As many patients in clinic are older, frailer, and/or co-morbid, it is likely that many patients in clinic with T4 disease may elect, weighing up risk and benefit, not to have treatment once confirmed to be ctDNA negative.

## Study design and statistical concerns

We would refute the statement by Olivier and Prasad that “ctDNA was not considered a high-risk feature before the study was run,” rather this was the justification for the DYNAMIC study, specifically the unprecedented RFS hazard ratio of 28 seen in a multivariate analysis of an initial observational cohort of stage II patients [7].

Olivier and Prasad highlight some of the challenges associated with designing non-inferiority studies, including defining non-inferiority margins, which will inform the statistical plan and sample size calculations. However, contrary to what they suggested, the justification for the non-inferiority margin was included in the DYNAMIC study manuscript [4]. Also, while a very small non-inferiority margin is desirable, this may be impractical. Larger non-inferiority margins may not preclude a practice-changing result. For example, the IDEA meta-analysis [8] failed to demonstrate non-inferiority of 3 versus 6 months of adjuvant chemotherapy, despite enrolling 12,834 patients. However, the IDEA study now informs all major practice guidelines [9], as the absolute difference in 3-year DFS was not clinically significant at less than 1%. It is worth noting also that the DYNAMIC study was positive not due to the size of the non-inferiority margin, as the RFS of ctDNA-informed patients was numerically higher than control patients. So the study would still have been positive even if a much tighter margin had been used.

## A response to the “Possible solutions” as suggested by Olivier and Prasad

We have concerns with their several suggested alternative study designs. First, they suggest any de-escalation strategy should be investigated as a combination of de-escalation and conventional care. Such a “de-escalation strategy” would necessarily result in more patients being treated in the intervention arm (i.e. would then be an escalation strategy), by adding any otherwise untreated biomarker (e.g. ctDNA) positive patients to the current pool of over-treated patients. Also, such a study design would need to be powered for superiority, an unrealistic goal in stage II colon cancer where no survival benefit from chemotherapy has been demonstrated when all patients are treated with chemotherapy. Another suggestion is that to “tackle the issue raised by non-inferiority margins”, “test based decision strategies should be superiority instead”. Again, this precludes any study trying to address current overtreatment.

Next, they suggest we should “limit de-escalation to settings where the current practice benefits have a widespread agreement”, which would necessarily preclude any clinical scenarios where there is marginal treatment benefit and where therefore patients are at the greatest risk of overtreatment, such as stage II colon cancer. Later they suggest such a superiority study “in stage III colon cancer, among those not receiving chemotherapy based on current management”. This is a perplexing suggestion, given that chemotherapy is standard of care (i.e. there is widespread agreement), where the only patients not currently treated are those considered not fit for treatment. So, the proposed study population as such does not exist?

## Conclusions

The National Academy of Medicine defined clinical utility of a cancer biomarker as “evidence that the strategy is either superior to standard of care or that is equivalent to standard of care with some other advantage”. This standard has been met by ctDNA in the DYNAMIC study, with many more ctDNA-informed trials underway, which will further refine our understanding of this promising biomarker. No clinical trial design is perfect, and any research effort can be criticised, but ultimately we need to conduct the randomised clinical trials that move us closer to the holy grail of personalised healthcare, including reducing treatment of patients very unlikely to benefit and ensuring the patients most likely to benefit do receive therapy.

## Data Availability

Not applicable as no original data is presented.

## Abbreviations

ctDNA: Circulating tumour DNA
RFS: Recurrence-free survival
